# Editorial: Bioinspired superwettable materials from design, fabrication to application

**DOI:** 10.3389/fbioe.2023.1204607

**Published:** 2023-04-28

**Authors:** Jingxin Meng, Hongliang Liu, Jun-Bing Fan, Pengchao Zhang, Tailin Xu, Feilong Zhang

**Affiliations:** ^1^ Technical Institute of Physics and Chemistry, Chinese Academy of Sciences (CAS), Beijing, China; ^2^ Binzhou Institute of Technology, Weiqiao-UCAS Science and Technology Park, Binzhou, China; ^3^ School of Chemistry and Chemical Engineering, Yantai University, Yantai, China; ^4^ Shandong Laboratory of Yantai Advanced Materials and Green Manufacturing, Yantai, China; ^5^ Southern Medical University, Guangzhou, China; ^6^ State Key Laboratory of Advanced Technology for Materials Synthesis and Processing, School of Materials Science and Engineering, Wuhan University of Technology, Wuhan, China; ^7^ Shenzhen University, Shenzhen, China; ^8^ Nanyang Technological University, Singapore, Singapore

**Keywords:** antifouling, biosensing, bioseparation, specific adhesion, bioinspired

Due to the combination of surface micro/nanostructure and surface chemical modification, superwettable interfacial materials have exhibited remarkable functions in muti-fields, such as anti-fouling, sensor detection, materials manufacture and medical treatment. The most advantageous strategy to construct and prepare superwetting materials and expand their probable applications is deeply investigating the potential mechanisms of superwetting biological organisms. During the last 2 decades, superwetting biological organisms with superhydrophobicity, superhydrophilicity, directional liquid-transportation, and multifunctional composite surfaces integrating superwettability with other physicochemical properties have brought great enlightenment to the development of superwettable materials. With further research of more unique biological phenomena, more practical superwetting materials will be utilized for a wider range of applications in the near future.

The primary task of this Research Topic is to compile high-level researches on bioinspired superwettable materials for the solution of actual scientific problems. In this Research Topic, we present ten original articles and seven review articles, aiming to highlight the recent progress of bioinspired superwetting materials in the fields as diverse as controlled preparation of functional materials, anti-fouling, biosensing and biomedicine, etc.

The deposition of unwanted objects (e.g., ice, wax and bacteria) has been caused significant Research Topic in both daily life and industrial production. As a result, there has been an increased interest in the development of superwetting interfacial materials that offer efficient and sustainable resistance to deposition. Zhang et al. prepared a cheap superwetting micro/nanostructured surfaces with excellent anti-icing properties and promising applications scenarios in low-temperature environments. The surface was fabricated by the combination of deep reactive ion etching, glancing angle deposition, and fluorocarbon deposition. Based on classical heterogeneous nucleation theory, Li et al. roughened and fluorization-modified an Al substrate for preparing the superhydrophobic surfaces. The water vapor condensation experiment finally confirmed that only superhydrophobic surfaces with coral-like micro/nano-structures showed excellent anti-condensation properties, the droplets appear slowly and the number of droplets is rare. Due to a higher nuclear barrier caused by the smaller nanostructure, most of the superhydrophobic materials areas remained dry. This research offers a new avenue for the practical application of advanced superhydrophobic materials in anti-solid and anti-liquid fouling. Biofilms, which are the primary cause of most oral diseases, originate from the attachment of salivary proteins and pioneer bacteria. Natural antifouling surfaces inspire new antibacterial strategies. Zhang et al. summarized the mechanisms and fabrication strategies of bio-inspired superwetting materials to prevent the adhesion of bacteria, and highlighted their applications in dentistry. These novel strategies provide a solid foundation for oral antimicrobial application and improving the efficacy of anti-bacteria. The reason why bio-inspired superhydrophobic preparation means has received intense attention in recent years is that it has been widely applied in anti-fouling, liquid-liquid separation, and other applications. Ge-Zhang et al. expounded the basic principle of superhydrophobic surface through different superhydrophobic models, summarized the structural features of biological superhydrophobic surfaces (e.g., lotus leaves), and detailly introduced the characteristics differences and applications of various surfaces. Finally, the challenges and future development directions of bionic superhydrophobic surfaces were discussed. However, the poor durability of bio inspired superhydrophobic materials limits their practical application. In addition to elucidating five typical superhydrophobic models, Luo et al. summarized the improvement of superhydrophobic surfaces in terms of wear resistance and chemical corrosion resistance, and discussed the testing measure of durability such as tape-peeling methods and electchemical corrosion. They also demonstrated the application of stable superhydrophobic interfacial materials in anti-fouling, mixture separation, membrane distillation, and electrochemical process.

With the advancement in the field of nanotechnology, nanomaterials or bionic nano platforms of different scales have been applied in related fields such as reverse electrodialysis, clinical analysis, etc. These materials have selective separation and recognition functions. Inspired by the electric eel, ions can be selectively transferred by their unique ion channels for generating electricity, Yao et al., performed a composite membrane based on metal-organic framework, thereby achieving high-effective power production from salinity difference of sea water and river water. The composite membrane has a dense structure and exhibits long-term stability in saline. This study provides a guiding path for producing the high-effective salinity-gradient power generation systems based on selective transportation of anion. To effectively separate phosphopeptides and glycopeptides, Shang et al. constructed silica microspheres modified with polyhistidine. The combination of hydrophilic and hydrogen bonding interactions endow silica microspheres with high selectivity and coverage, benefiting for the Research Topic of phosphopeptides and glycopeptides at the same time, . Furthermore, this strategy allows sequential elution of phosphopeptides and glycopeptides, showing significant potential in co-analysis of protein in clinical medicine. For the patients with chronic kidney disease, cardiac surgery-associated acute kidney injury (CSA-AKI) may increase the mortality rates of the disease. Bai et al. used Gemini C_18_ column and high-resolution mass spectrometry to analysis the proteomic of urine samples from six CSA-AKI patients, aimed to investigate the possible correlation between changes in urine proteomics and CSA-AKI. The Gemini C_18_ silica microspheres can be enhanced the protein recognition rate to achieve highly precious resources for the urinary differential expressed proteins of AKI. This analysis provides indispensable foundation about urinary proteome biomarkers and valuable resources for deeper study of AKI. Additionally, Wu et al. concluded the various bio-inspired nanoparticles (e.g., metallic nanoparticles, polymeric nanoparticles and nanovesicles) in biomedical fields and discussed the progress of bioinspired nanotechnology in biomedicine. Then, they highlighted the importance of fabricating nanoparticles through the bioinspired route. Finally, the preparation of new nanoparticles and their applications in the field of biomedicine are prospected.

Superwettable surfaces have also been extensively studied for use in fabricating sensors (e.g., electrochemical immunosensor and non-enzymatic sensors) in medical field. As one of the neurodegenerative disease, Alzheimer’s disease (AD) is caused by the injury of brain neurons, which severely affect human normal life and health. Based on the superwetting microdroplet array, Huang et al. reported an sensing platform by electrochemical way for detecting various AD biological markers in blood. In comparison, this superwetting sensor has excellent properties such as large specific surface area, excellent conductivity and prominent biocompatibility. In addition to health detection, Chen et al. developed a non-enzyme sensor with the liquid-solid-air triphase interfacial electrode for electrochemical applications. The sensor collaboratively utilizes the property of electrocatalytic glucose oxidation to promote the formation of local alkalinity production. The high local pH value is obtained through the oxidation reaction at the three-phase interface, thus realizing the electrochemical detection of glucose at neutral solution. For acquiring deep insight into the biosensors, Yang et al. clearly introduced the sensing methods of superwetting biosensors for disease detection by biomarkers, which mainly introduces disease analysis by fluorescence analysis, electrochemistry display, surface enhancement Raman scattering assay and visional means. The author further systematically introduces the applications of super-wettable biosensors in the field of biomarkers, and finally gives suggestions on the future challenges and development of sensors.

As one of the superwettable materials, interestingly, superwetting materials can enhance the ability of cartilage regeneration. Inspired by the mussel-adhesive phenomenon, Chi et al. proposed a simple preparation method for osteoconductive and osteoinductive nanomaterials utilizing material extrusion techniques and surface modification strategies. By adding polydopamine and hydroxyapatite nanoparticles on the surface of the composite material, the 3D printed porous scaffold with enhanced osteogenesis was prepared. The physical and chemical properties of the scaffold such as surface wettability, roughness, mechanical performance, and biodegradability was studied to demonstrate the enhanced osteogenesis ability. The superwetting material will inevitably endure the impact of the bone, Liu et al. investigated the impact resistance and energy dissipation of multilayer bioinspired composites based on the fiber periodic helical structure of fibers. Under the same material component and property parameters, adjusting the fiber spiral angle of the fiber can effectively improve the stress concentration of the bioinspired materials caused by external impact. In conclusion, the mineralized collagen fibers based on the periodic spiral structure in osteons can effectively improve the impact resistance property of cortical bone. The research results have guiding significance for the design and preparation of high performance biomimetic osteogenic superwetting materials. Inducing cartilage reproduction can cure temporomandibular disorders with biomaterials. However, the wettability of bone-filled biomaterials was not satisfactory. For addressing this problem, Yang et al. placed mesenchymal stem cells with wetting properties on the surface of TGF-β-loaded gelatin methacryl microspheres, resulting in active wetting of biomaterials. Modified gelatin-MSCs microspheres can more effectively localize bone defect repair sites, expediting the healing of temporomandibular joint defect area caused by releasing cytokines at specific sites. This method provides a new strategy for the development of cartilage regeneration materials through the addition of infiltrating factors. Therefore, superwetting materials will play a great role in bioengineering and medical remediation. As one of the bioinspired superhydrophilic materials, hydrogels have excellent properties such as biocompatibility, biodegradability and strong crosslinking. The above excellent physical and chemical properties will make hydrogels promising to be a new delivery platform and unconventional therapy for repairing endometrial damage. Many works on bioinspired-hydrogels for subcavity endometrial repair was discussed. For example, As the post-operative physical barrier and therapeutic delivery platform, Dong et al. discussed recent developments in hydrogel delivery platforms for endometrial repair. In addition, the development status, application limitations and future development of hydrogels are discussed in detail. Liquid-infused surfaces (LIS) also have unique prospects in the fields of biological engineering, medical equipment, and biosensor. LIS also play an important role in the fields of bioengineering, medical devices and biosensing. Yang et al. focused on the influence of liquid layers on the properties of medical materials. At the same time, the development trend of information system in the future is forecasted.

Overall, this Research Topic cover several cutting-edge fields in bio-inspired materials with superwettability, such as anti-adhesive materials, sensing detection systems, life medical treatments, etc., which help readers understand the application progress of bio-inspired materials with superwettability. Despite the encouraging results mentioned above, more research is still needed to gain a deeper understanding of the mechanisms for applications and to develop more superwetting materials that can be applied more quickly.

